# CNR1 and CNR2 Cannabinoid Receptor Mutations in Cancer Cells

**DOI:** 10.3390/cimb48060610

**Published:** 2026-06-11

**Authors:** Lillian Schneider, Maria Ruano, Camryn R. Mackey, Kiersten Spiegel, Renee A. Bouley, Ruben C. Petreaca, Ryan J. Yoder

**Affiliations:** 1 Biology Undergraduate Program, The Ohio State University, Marion, OH 43302, USA; schneider.1303@buckeyemail.osu.edu (L.S.); ruano.9@buckeyemail.osu.edu (M.R.); mackey.246@buckeyemail.osu.edu (C.R.M.); spiegel.108@osu.edu (K.S.); 2 Department of Chemistry and Biochemistry, The Ohio State University, Marion, OH 43302, USA; bouley.8@osu.edu; 3 Department of Molecular Genetics, The Ohio State University, Marion, OH 43302, USA; 4 Cancer Biology, The James Comprehensive Cancer Center, The Ohio State University, Columbus, OH 43210, USA

**Keywords:** cannabinoid receptors, cancer, genetic mutation

## Abstract

Cannabinoids, including the psychoactive D9-tetrahydrocannabinol (THC) and the non-psychoactive cannabidiol (CBD), interact with receptors within the endocannabinoid system. The major receptors within this system are *CNR1* (cannabinoid receptor 1) and *CNR2* (cannabinoid receptor 2), which are both seven-transmembrane G-protein-coupled receptors. In this report, we used the Catalogue of Somatic Mutations in Cancers (COSMIC) to map and analyze mutations arising in CNR1 and CNR2. The goal was to determine if any trends or signatures could be identified. We identified several mutations in both *CNR1* and *CNR2*. In silico 3D structure of proteins reveals that these mutations cluster on the intracellular regions of *CNR1* and *CNR2*, and certain residues may be able to destabilize the interaction with the G-alpha protein due to their close proximity. mRNA expression showed that *CNR1* and *CNR2* are within normal expression levels in most cancer types except kidney, where there is a tendency towards over-expression. Neither *CNR1* nor *CNR2* is a driver gene, and our analysis shows that mutations in cancer cells are deactivating (e.g., loss of function).

## 1. Introduction

D9-tetrahydrocannabinol (THC) is the psychoactive cannabinoid produced by three species of the genus Cannabis. Of these, *Cannabis sativa* and *Cannabis indica* yield the most concentration of THC [1,2]. In addition to THC, the plants produce several other molecules including cannabidiol (CBD), which has almost no psychoactive effects but has been reported to possess some therapeutic properties [3]. For many years, cannabinoids have been used as recreational drugs as well as pain relievers and mood modulators for various diseases, including cancer [4]. Both THC and the related compounds also have anti-inflammatory properties [5,6], which may be one reason they are used, at times, as therapeutic agents.

THC and related compounds can be either agonists or antagonists of receptors within the endocannabinoid system, primarily with roles in nervous system development. Cannabinoids also function in regulating appetite, pain, mood, as well as certain developmental processes, including fertility and pregnancy [4,7]. Two major cannabinoid receptors are *CNR1* (cannabinoid receptor 1) and *CNR2* (cannabinoid receptor 2), also known as *CB1* and *CB2*. Both *CNR1* and *CNR2* are seven-transmembrane G-protein-coupled receptors (GPCRs) [8,9]. Upon activation, they inhibit adenylyl cyclase, leading to a decrease in cAMP levels [10,11,12] and activate the mitogen-activated protein kinase (*MAPK*), a function modulated primarily by *CNR1* [13]. Of the two major cannabinoid compounds, THC acts as an agonist while CBD acts as an antagonist of *CNR1* and *CNR2*. When classified by structural homology, *CNR1* and *CNR2* are closely related within the GPCR families. They are in the same branch as the adenosine A1 receptor (*ADORA1*) [14] and the related *ADORA2* and *ADORA3* [15] which also have functions in adenylyl cyclase regulation and fertility.

Several levels of evidence suggest that THC and related compounds have anticancer effects by inducing apoptosis, arresting the cell cycle, and inhibiting metastasis and angiogenesis, among other functions [16,17,18,19]. Early experiments have shown that the CBD antagonist can inhibit the proliferation of glioblastoma cells [20,21]. Similarly, THC has also been shown to both inhibit proliferation and kill glioblastoma cells [22]. THC appears to act by inhibiting the *RAS-MAPK* and *PI3K-AKT* pathways involved in cell survival [17,22,23]. Additionally, THC and CBD have been shown to synergize with other anticancer therapeutic agents [24,25,26,27]. Conversely, at least a couple of reports have shown that THC may promote tumor growth [28,29]. Thus, the roles of cannabinoids in cellular proliferation and cancer progression are complex.

Certain cannabinoid receptor mutations affect both ligand interactions and downstream signaling [30,31]. In this report, we characterized somatic CNR1 and CNR2 mutations in cancer cells using the Catalogue of Somatic Mutations in Cancers (COSMIC) database [32]. The goal was to obtain a pan-cancer map of *CNR1* and *CNR2* mutations. Additionally, we characterized gene expression profiles of *CNR1* and *CNR2* in all reported cancer tissues.

## 2. Materials and Methods

### 2.1. Data Acquisition and Analysis

Complete mutation data for *CNR1* and *CNR2* were downloaded from the Catalogue of Somatic Mutations in Cancer (COSMIC) as a .csv file (Version 99) [32]. Data were analyzed in Excel or SPSS (version 31). Graphs were generated with either Excel or SPSS.

### 2.2. Overexpression and Copy Number Analysis

Gene expression data were downloaded from COSMIC for TCGA samples. COSMIC reports expression data as Z-values, which are normalized to non-cancer tissue. A Z-value between −2 and +2 is considered normal expression, while over +2 is over-expressed and under −2 is under-expressed [33]. Allele copy number was extracted from COSMIC using the copy number analysis tool (CONAN, version 2.28, https://cancer.sanger.ac.uk/cosmic/conan/search, accessed on 2 August 2025) [34].

### 2.3. cBioPortal Statistics

To calculate *CNR1* and *CNR2* co-occurrence statistics, we used the cBioPortal TCGA PanCancer Atlas Studies [35,36,37]. Mutual exclusivity *p*-values and Log2 odds were calculated for the *CNR1* and *CNR2* pair.

### 2.4. Protein Structure Analysis

PDBePISA (http://www.ebi.ac.uk/pdbe/prot_int/pistart.html (accessed on 2 August 2025)) is an online system that evaluates different proteins, interfaces, structures, and assemblies [38]. The software was used to identify interactions that occur between protein structures within a PDB file. Salt-bridges, hydrogen bonds, and other interactions were mapped within the system. PyMOL is a computer software system that can be used to view and analyze molecular structures. Features within PyMOL allow for the identification of residue locations and interactions between residues in a protein structure. This software also has the capability of administering mutations to observe their effects. The PyMOL Molecular Graphics System, Version 2.0, Schrödinger LLC, was used for all electrostatic analysis.

All figures were made in Photoshop.

## 3. Results and Discussion

### 3.1. Mutation Spectra of CNR1 and CNR2

**Figure 1:**
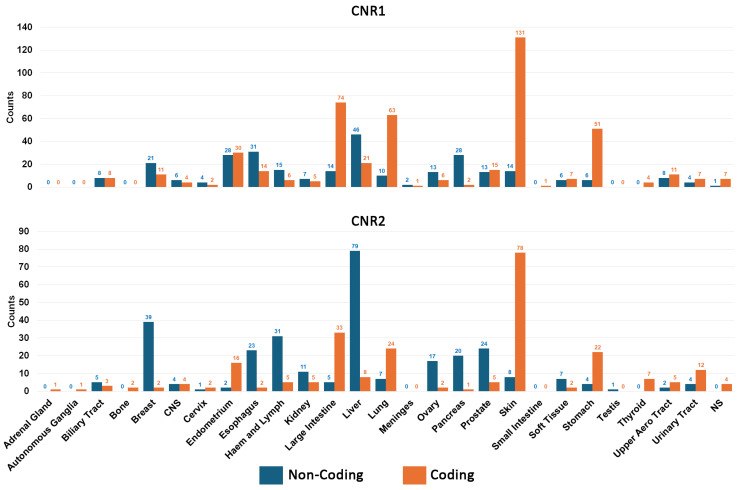
Distribution of *CNR1* and *CNR2* mutations in all cancer tissues. The samples reported on COSMIC were partitioned into coding and non-coding. The top panel shows the distribution of these mutations for *CNR1*, and the bottom panel shows the distribution for *CNR2*. The numbers on top of the bars indicate the sample counts for each tissue in each category.

COSMIC reports both protein-coding and non-coding (intronic and 5′ and 3′ UTRs) mutations. All queried cancer tissues acquire mutations in both coding and non-coding regions for both genes (Figure 1). The skin is remarkable for having a much higher ratio of coding to non-coding mutations for each gene. Coding mutations change the sequence of protein amino acids and are likely to affect the function of the gene. One interpretation is that the high frequency of coding mutations in skin suggests that destabilizing their function may promote skin cancer, but the data ought to be interpreted with caution (see below). Other tissues characterized by a higher ratio of coding to non-coding mutations are the large intestine, lung, and stomach. *CNR2* also shows an increase in the coding-to-non-coding ratio, but these data should also be interpreted with caution because there are only a few samples. For tissues with even fewer sample numbers (e.g., adrenal gland, bone, etc.), we are hesitant to make any interpretation because there are very few data points.

Conversely, breast, liver, esophagus, hematopoietic and lymphoid, ovary, pancreas, and prostate cancers have more *CNR2* non-coding than coding mutations, suggesting that coding mutations may not be tolerated in these tissues. An incidence of higher non-coding to coding mutations is also found for *CNR1* in breast, liver, esophagus, ovary, and pancreas. Other tissues like *CNR1* ovary and *CNR2* kidney also have different numbers for coding and non-coding, but the ratio is lower, and any interpretation would not be as strong as that for skin or liver. Again, no interpretation can be made for tissues with few data points (e.g., meninges, testis, etc.). Thus, it appears that mutation distributions in these two genes are similar in some cancers but different in other cancer tissues, highlighting the unique functions of these receptors.

We next partitioned all coding mutations into missense, nonsense, silent, InDels (insertions and deletions without frameshifts), and complex (insertions and deletions with frameshifts leading to truncations) (Supplementarys Figure S1, Table S1). We wanted to get a sense of the frequency of detrimental mutations appearing in these two genes. For example, nonsense mutations truncate parts of the protein and are predicted to be more deleterious than silent mutations that do not change the protein sequence. Missense mutations were the most abundant and appeared in nearly every cancer tissue sampled, with some exceptions. For example, the adrenal gland does not have *CNR1* missense mutations. In fact, no adrenal gland mutations are reported on COSMIC for *CNR1*. This is most likely because the sample size is too small (three samples). Non-sense and other out-of-frame insertions were identified in both genes, but only in certain cancer types (Supplementary Figure S1). Finally, silent mutations were found in most cancer types.

We paid particular attention to skin, which has a high ratio of coding-to-non-coding mutations. However, almost half of the coding mutations are silent (45.8% for *CNR1* and 47.4% for *CNR2*). Silent mutations do not change the function of proteins/enzymes, and in fact, a similar number of silent to non-silent mutations is a strong indication of neutral selection [39]. Thus, the safest interpretation here is that although skin shows a higher ratio of coding to non-coding, this may simply be due to a large number of samples deposited for skin cancers and not necessarily an indication of selection in skin.

**Figure 2:**
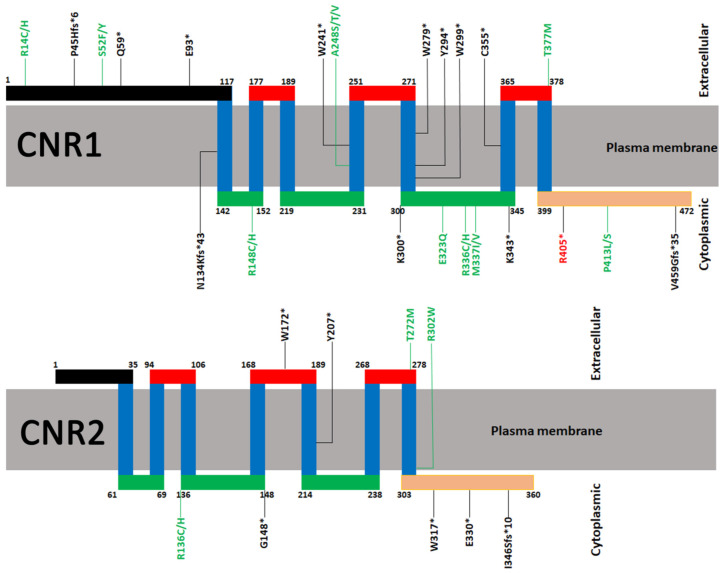
Truncating and high-frequency mutations. Cartoon models of *CNR1* and *CNR2* showing the position of truncating (black) and high-frequency point mutations (green). Truncating mutations are frameshifts that will result in a stop codon downstream (e.g., P45Hfs*6 has a frameshift at amino acid 45, which results in a stop codon six amino acids downstream) or nonsense (e.g., Q59*). For high-frequency point mutations, all amino acid substitutions at a certain location identified on COSMIC are shown (e.g., R14C/H indicates that R14 is sometimes substituted with C and other times with H). The major *CNR1* recurring mutation (R405*) is shown in red.

To understand where these mutations map on CNR1 and CNR2 protein sequences, we produced cartoon structures of proteins showing the various domains (Figure 2). As G-protein-coupled receptors, *CNR1* and *CNR2* are characterized by seven transmembrane domains. *CNR1* has longer extracellular N-terminal and cytoplasmic C-terminal domains, while *CNR2* is characterized by shorter N-terminal and C-terminal domains. The transmembrane domains are about the same size for both proteins.

Ligand binding occurs in the N-terminal domain while the C-terminal domain interacts with G-proteins to transduce the signal. The interaction of the *CNR1* C-terminus with G-proteins and activation of downstream signals is highly regulated. Interaction of beta-arrestin2 with the C-terminus results in receptor internalization [30]. Conversely, interaction of beta-arrestin1 with the C-terminus causes G-protein independent signaling. *GRK3* phosphorylates several residues in the C-terminus to modulate these interactions. Importantly, C-terminal truncations (removing sequence from residue 417) cause constitutive activation of the G-protein signaling pathway [40,41]. We identified a recurring truncation in this region (R405*) (Figure 2). Mutation hotspots have been proposed to occur when they appear in five samples or more [42,43,44]. The R405* “hotspot” was identified in 10 samples: five large intestine, one liver, one stomach, one biliary tract, one endometrium, and one hematopoietic and lymphoid tissue [45,46,47,48,49]. Although a pan-cancer analysis shows that this mutation could be classified as a hotspot, most identified tissues have only one mutation. Therefore, we interpret these results with caution, as it does not appear that this mutation drives any specific cancer. Truncations in other regions have also been identified, but are likely to completely inactivate the gene and may not behave as dominant (Figure 2).

*CNR2* has a shorter extracellular N-terminus and intracellular C-terminus (Figure 2). Several C-terminal truncations occur in *CNR2*, which also remove the cytoplasmic region. *CNR2* signals through a Gi/0 protein to inhibit adenyl cyclase, but this interaction appears to occur between Gi/0 and residues in the cytoplasmic loops, with minor contributions of the C-terminus [50,51,52]. However, the C-terminus regulates receptor function through interaction with various modulators, including beta-arrestin [53]. Phosphorylation of S352 is required for receptor desensitization and internalization [54]. Thus, these C-terminal truncations have the potential to also constitutively activate *CNR2*.

Mutations leading to a truncation (e.g., nonsense and frameshift) have a drastic effect on protein function and often on expression levels. Unfortunately, COSMIC only reports mRNA expression (e.g., transcription) for TCGA samples but not protein expression (translation) or stability. For non-TCGA samples, not even mRNA expression is reported. We looked at mRNA expression levels for truncated alleles, and they are within normal ranges (Supplementary Table S2). Thus, it appears that truncations do not change transcription, but no conclusion can be made about translation or protein stability from the available data.

### 3.2. Co-Occurring CNR1 and CNR2 Mutations

We identified 17 samples that have mutations in both *CNR1* and *CNR2* (Supplementary Table S3). Four of these samples have a high-frequency mutation in either *CNR1* or *CNR2*. A skin adnexal tumor sample (*CNR1* p.S52Y; *CNR2* p.D24H) [55] was isolated from a Central European patient. Other demographics are not known for this sample. Another skin sample (*CNR1* p.Q97K; *CNR2* p.R302L, p.W172L) was isolated from malignant melanoma of an 80-year-old male. One endometrial cancer sample (*CNR1* p.F381L; *CNR2* p.R136C, p.S60P) was isolated from a 33-year-old female with endometrial carcinoma. Finally, one stomach sample with high mutation burden in *CNR2* (*CNR1* p.W255R; *CNR2* p.F200I, p.I156V, p.G155C, p.T153A, p.L145V, p.S140T, p.I129V) is from a cultured sample of a 66-year-old female. The sample was isolated from adenocarcinoma of the gastroesophageal junction [56]. These data show that destabilizing both *CNR1* and *CNR2* in cancer tissues, although rare, is possible. Indeed, co-occurrence statistical analysis using the cBioPortal mutual exclusivity calculator shows a Log2 odds greater than three and a *p*-value lower than 0.001, indicating a tendency towards mutation co-occurrence between *CNR1* and *CNR2*.

### 3.3. Expression and Copy Number Profiles of CNR1 and CNR2

**Figure 3:**
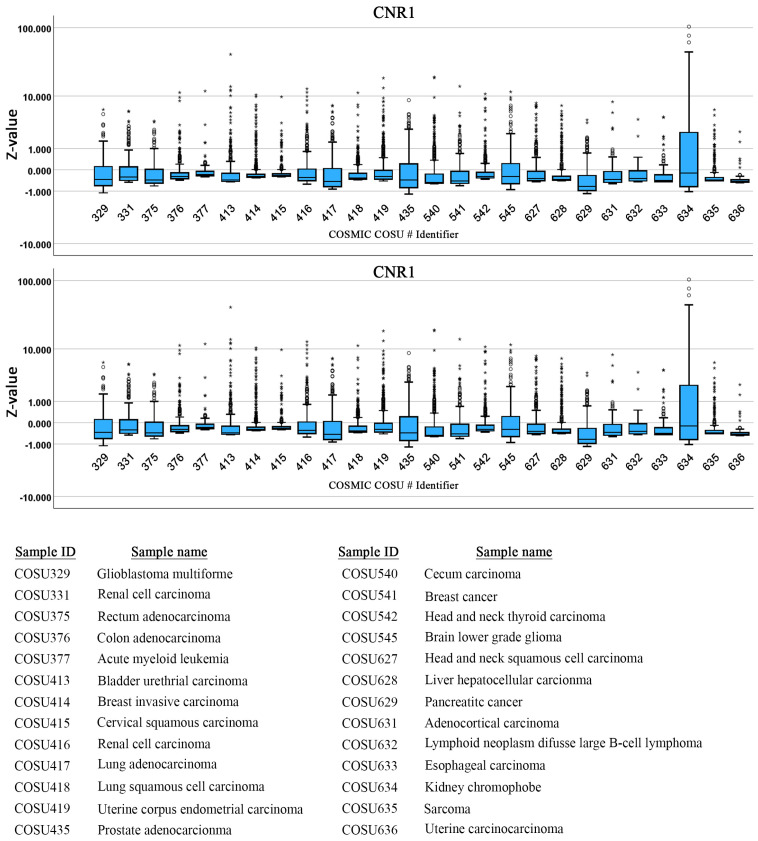
mRNA expression levels parsed by cancer type. Only TCGA samples report mRNA expression. The X-axis shows the COSU identifying number (#). The legend below the graphs correlates the study identifier number with the cancer type. The star and dots represent outliers outside of the 95% confidence interval.

Overexpression of *CNR1* and *CNR2* has been reported in many cancers [57,58,59,60]. Expression data is available for TCGA samples and COSMIC reports data for 9144 *CNR1* and CNR2 samples. Note that most of the samples for which expression profiles are available do not have *CNR1* and *CNR2* mutations, which explains why the total number of samples is higher. Of these, 321 *CNR1* (3.5%) and 215 *CNR2* (2.35%) samples show mRNA over-expression. TCGA data is presented as Z-values, which are normalized to non-tumor tissue (see Section 2.2). We partitioned the samples by cancer type (Figure 3). This analysis shows that most cancers show expression within the normal range (−2 > Z > +2) with a few outliers with a tendency towards over-expression. Kidney cancers are the exception. Although the mean of all the samples is within normal expression, the confidence interval spreads above +2 for both *CNR1* and *CNR2*. The significance of this finding is not immediately obvious, but it may indicate that higher expression of *CNR1* and *CNR2* promotes tumor development in a subset of cancers.

We next checked copy number alterations in *CNR1* and *CNR2* (Supplementary Table S4). Copy number alterations were reported for 46 *CNR1* samples but only 11 *CNR2* samples. Although there is a general tendency towards high copy number (e.g., amplification) rather than loss of heterozygosity or homozygous deletion, there is no correlation between this and cancer type. Rather, samples showing amplification occur in multiple types of cancers. Further, only eight samples were reported for *CNR2*. Taken together, these data show that although an increase in copy number may be driving some tumors, it is premature to make this general conclusion based on a limited sample pool.

### 3.4. Potential Interactions of Frequent Mutations with G Alpha

**Figure 4:**
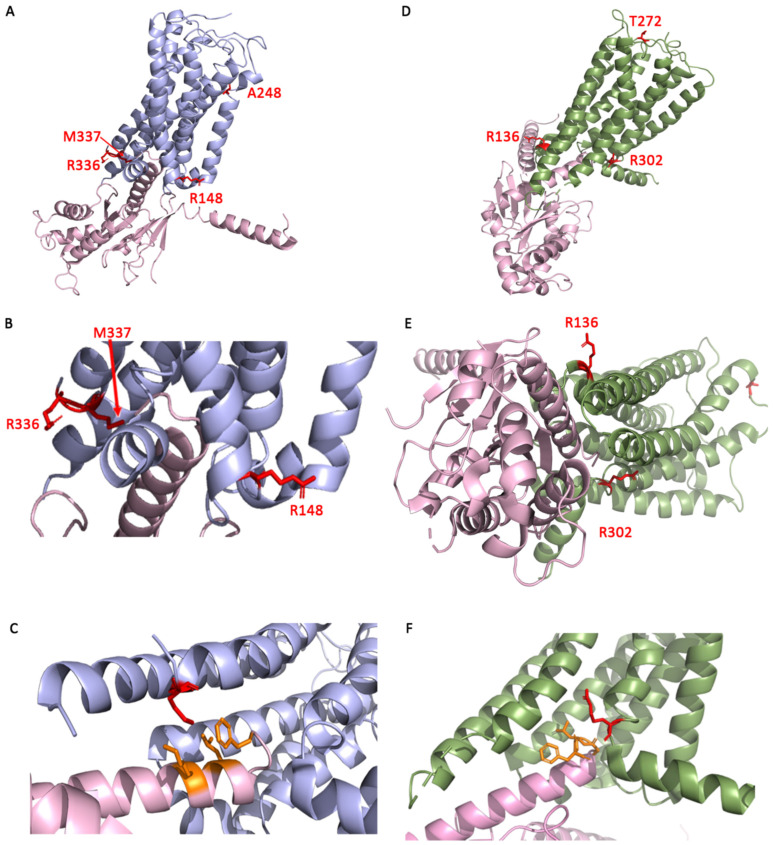
In silico structures of the CNR1 and CNR2 receptors showing the position of high-frequency mutations. (**A**). CNR1 receptor (purple) interacting with G alpha (pink). The residues shown in red are the frequent mutations of CNR1 identified from COSMIC. (**B**). A zoomed diagram is shown in (**A**) to show the hotspots that were located near where G alpha binds to CNR1. (**C**). Residue M337 (red) of the CNR1 receptor (purple) was found to interact with residues K345, L348, and F354 (orange) of the G alpha protein (pink). These nonpolar interactions are assumed to be due to their proximity being within 4 Å of M337 of CNR1, as determined through PyMOL. (**D**). CNR2 (green) interacting with G alpha (pink). Shown in red are the reported frequent mutations of the CNR2 gene. (**E**). An intracellular view of the CB2-G alpha binding complex to show the proximity of the mutation hotspots R136 and R302 to G alpha. (**F**). A closer look at the interaction of R302 (red) of CNR2 with residues G152, L153, and F154 (orange) of G alpha (pink). These interactions were mapped using PyMOL to find interacting residues within 4 Å of R302.

From the *CNR1* COSMIC data, we identified 17 frequent mutations occurring at nine different locations: p.R14C/H, p.S52F/Y, p.R148C/H, p.A248S/T/V, p.E323Q, p.R336C/H, p.M337I/V, p.T377M, and p.P413L/S. When looking for these mutation locations on the Cryo-EM structure of the human CNR1-G protein complex (PDB: 6KPG) [61], we were able to locate four of the nine mutations. Residues R148, A248, R336, and M337 of *CNR1* were a part of the Cryo-EM sequence. Of these four residues, R148, R336, and M337 all appeared closer to the intracellular portion of the *CNR1* receptor site (Figure 4A,B). Residue A248 is located further away from the binding site, closer to the extracellular portion of *CNR1*. The remaining residues were unable to be mapped on our receptor structure due to the residues not being included in the identified Cryo-EM sequence. The absence of these sequence portions is likely due to large amounts of movement in these segments, which makes it hard to accurately identify the amino acid sequence using Cryo-EM technology.

When the CNR1-G alpha protein-binding complex was analyzed through PDBePISA, it was confirmed that the mutation hotspots at R336 and M337 were likely interacting with G alpha. We utilized PyMOL to identify any residues within 4 Å of our frequent mutations to see if the software could detect which residues were interacting with G alpha in any way. Of the three mutations that were located near the G alpha binding site, only M337 was found to have G alpha residues within 4 Å of its location (Figure 4C). These G alpha residues—K345, L348, and F354—are presumed to be interacting with M337 due to their proximity.

From the COSMIC data, CNR2 was found to have five frequent mutations occurring at three different locations: p.R136C/H, p.T272M, and p.R302L/W. When looking for these mutation locations on the Cryo-EM structure of the human CNR2-G protein complex (PDB: 8GUQ) [62], we were able to locate all three of the mutations. Residue T272 is in the extracellular portion of CNR2 (Figure 4D). This mutation is closer to the ligand active site of CNR2, but is not a residue that has been identified to play a role in agonist or antagonist binding. Residues R136 and R302 are located towards the intracellular side of CNR2 (Figure 4D,E).

When the CB2-G protein-binding complex was analyzed with PDBePISA, it confirmed that the R302 mutation was interacting with G alpha. We utilized PyMOL to identify any residues within 4 Å of this mutation to see if the software could detect which residues were interacting with G alpha in any way. PyMOL detected R302 to be within 4 Å of residues from the G alpha protein (Figure 4F). These G alpha residues—G152, L153, and F154—are presumed to be interacting with R302 due to their proximity to the mutation. These residues on CNR1 and CNR2, within close proximity to G alpha, would be of particular interest for further study to see if their mutation affects the interaction that is central to the signal transduction.

### 3.5. Patient Information for Key Mutations

We wanted to dig deeper into sample information for the key mutations shown in Figure 2. COSMIC reports some demographics for some patients but not all (Supplementary Table S5). An inspection of these data shows that it occurs in both males and females with generally equal frequency. It also does not track with the type of treatment or behaviors (e.g., smoking, drinking, etc.). The age of diagnosis is higher (over 50), indicating that these mutations are due to biological age and probably arise due to the accumulation of DNA damage. Some data show that mutations arising in younger patients lead to more aggressive cancers than in older patients because they are more likely to concentrate in cancer driver genes [63,64,65], but this does not appear to be the case here. CNR1 and CNR2 mutations may contribute to tumor development, but most likely do not drive cancers.

## 4. Conclusions

The endocannabinoid system has been identified as potentially having a role in cancer development. Using cancer data deposited on COSMIC, we identified several *CNR1* and *CNR2* mutations that occur in multiple samples, which some reports characterize as frequent [42,43,44]. Through the analyses performed in this study, we highlighted the different changes that these mutations have on the endocannabinoid receptors. Most of the mutations that were identified are on the intracellular side of the receptors. Additionally, we find that mutations are generally destabilizing, most likely causing loss of function of *CNR1* and *CNR2*. Further, mRNA expression analysis of TCGA samples, which represent primary cancers, shows that *CNR1* and *CNR2* do not appear to be overexpressed in most cancers except the kidney. Although some samples within each cancer type do show increased expression, the general trend is normal expression. This was somewhat unexpected but not previously unreported in the literature. In fact, it appears that an increase in *CNR1* and *CNR2* expression may be associated with decreased colorectal cancer metastasis [66]. These findings are also significant in further elucidating the relationship between cannabinoids and the endocannabinoid system in the wider mechanisms of cancer and its treatment. Although all analyses here were in silico, our findings could inform experimental analysis and potential therapeutic avenues.

## Data Availability

Data used in this article are publicly available at COSMIC and cBioPortal. All analyses of protein structure are available in the article’s figures or Supplementary Tables and Figures.

## Supplementary Materials

The following supporting information can be downloaded at: https://www.mdpi.com/article/10.3390/cimb48060610/s1.
