# Supplementary material for: CNR1 and CNR2 Cannabinoid Receptor Mutations in Cancer Cells
Source: Curr Issues Mol Biol. 2026 Jun 11;48(6):610. doi: 10.3390/cimb48060610 (PMC13298140; doi:10.3390/cimb48060610)

Supplementary Figure S1. Tissue distribution of coding and non-coding mutations reported on COSMIC for CNR1 and CNR2. Coding and non-coding mutations were partitioned by cancer type for both CNR1 and CNR2 then the frequency of each type of mutation was graphed.

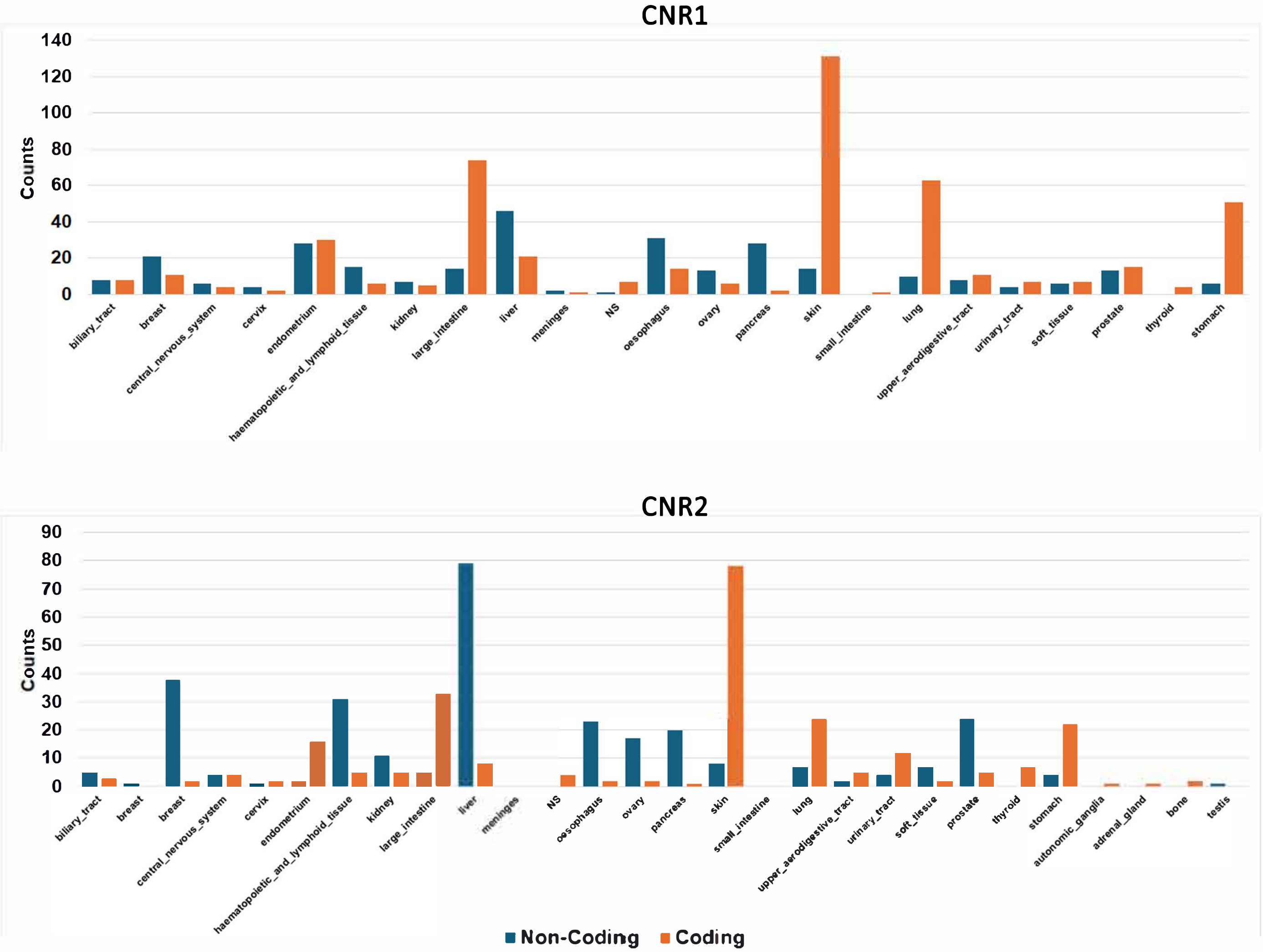

Supplement: Supplementary file 1 [file cimb-48-00610-s001.zip › Supplementary Figure S1.pdf]
