# Supplementary material for: CNR1 and CNR2 Cannabinoid Receptor Mutations in Cancer Cells
Source: Curr Issues Mol Biol. 2026 Jun 11;48(6):610. doi: 10.3390/cimb48060610 (PMC13298140; doi:10.3390/cimb48060610)
Supplement: Supplementary file 1 [file cimb-48-00610-s001.zip › Supplementary Table S2.pdf]

Supplementary Table S2. CNR1 and CNR2 mutation effects on gene expression.

Mutation	Sample name	Cancer type	CNR1 Z-value (F)
**CNR1**			
p.C355*	LUAD-5V8LT	Lung	N/A
p.E93*	TCGA-53-7626-01	Lung	-0.204
p.K300*	TCGA-78-8640-01	Lung	-0.578
p.K343*	TCGA-37-3792-01	Lung	-0.366
p.N134Kfs*43	TCGA-BR-4292-01	Stomach	-0.569
p.P45Hfs*6	TCGA-CF-A47Y-01	Urinary tract	0.098
p.Q310*	EXTERN_MELA_20140924_097	Skin	N/A
p.Q59*	Pat_08_A	NS	N/A
p.Q59*	Pat_08_B	NS	N/A
p.R405*	WGC003614	Biliary tract	N/A
p.R405*	CS1-C	Endometrium	N/A
p.R405*	SJALL043850-R	Hematopoietic and lymphoid tissue	N/A
p.R405*	MSU1-c	Large intestine	N/A
p.R405*	sysucc-1221T	Large intestine	N/A
p.R405*	T1755	Large intestine	N/A
p.R405*	TCGA-AD-5900-01	Large intestine	0.098
p.R405*	MSU1-a	Large intestine	N/A
p.R405*	Rk105_C01	Liver	N/A
p.R405*	GCTK_14274_T	Stomach	N/A
p.V459Gfs*35	Pat_24_A	NS	N/A
p.W241*	2492720	Skin	N/A
p.W241*	2492721	Skin	N/A

p.W241*	2492722	Skin	N/A
p.W241*	2492723	Skin	N/A
p.W241*	YUMER	Skin	N/A
p.W279*	PD42119a	Skin	N/A
p.W279*	TCGA-BF-AAP4-01	Skin	-0.544
p.W299*	MBC_45	Breast	N/A
p.W299*	2834130	Skin	N/A
p.Y294*	OSCC-GB_00770111	Upper aerodigestive tract	N/A
**CNR2**			
p.Y207*	CHG-97T	Liver	N/A
p.G148*	DU-145	Prostate	N/A
p.W172*	PD36792a	Skin	N/A
p.W172*	TCGA-Z2-A8RT-06	Skin	-0.194
p.W317*	C000-RCWM1J	Skin	N/A
p.E330*	AS-16	Soft tissue	N/A
p.I346Sfs*10	1117	Urinary tract	N/A
