# Supplementary material for: CNR1 and CNR2 Cannabinoid Receptor Mutations in Cancer Cells
Source: Curr Issues Mol Biol. 2026 Jun 11;48(6):610. doi: 10.3390/cimb48060610 (PMC13298140; doi:10.3390/cimb48060610)
Supplement: Supplementary file 1 [file cimb-48-00610-s001.zip › Supplementary Table S5.pdf]

Supplementary Table S5: Patient demographics for truncating mutations.

CNR1 mutations

Mutation	Patient number	Cancer type	Gender	Age	Other demographics
P45Hfs*6	TCGA-CF-A47Y-01	Urinary tract	Male	55	
Q59*	Pat_08_A	NS	Female	61	-Pretreatment<br>-Vemurafenib clinical partial response
Q59*	Pat_08_B	NS	Female	61	-Post-treatment<br>-Vemurafenib clinical resistant recurrence
E93*	TCGA-53-7626-01	Lung	Female	76	
N134Kfs*43	TCGA-BR-4292-01	Stomach	Female	73	
W241*	2492720	Skin	N/A	N/A	
W241*	2492721	Skin	N/A	N/A	
W241*	2492722	Skin	N/A	N/A	
W241*	2492723	Skin	N/A	N/A	
W241*	YUMER	Skin	N/A	N/A	
W279*	PD42119a	Skin	Male	51	
W279*	TCGA-BF-AAP4-01	Skin	Male	61	
W299*	MBC_45	Breast	N/A	N/A	
W299*	2834130	Skin	Female	59	

Y294*	OSCC-GB_00770111	Upper aerodigestive tract	Male	38	
K300*	TCGA-78-8640-01	Lung	Male	59	
K343*	TCGA-37-3792-01	Lung	Male	69	
C355*	LUAD-5V8LT	Lung	Male	52	-Asian<br>-Current smoker
R405*	WGC003614	Biliary tract	Male	66	-Chinese<br>-No alcohol intake
R405*	CS1-C	Endometrium	Female	84	
R405*	SJALL043850-R	Hematopoietic and lymphoid tissue	Female	12.84	-Chemotherapy clinical resistant recurrence
R405*	MSU1-c	Large intestine	Male	68	-Korean
R405*	sysucc-1221T	Large intestine	Male	64	
R405*	T1755	Large intestine	Male	74.83	
R405*	TCGA-AD-5900-01	Large intestine	Male	67	
R405*	MSU1-a	Large intestine	Male	68	-Korean
R405*	RK105_C01	Liver	N/A	N/A	
R405*	GCTK_14274_T	Stomach	Male	N/A	
V459Gfs*35	Pat_24_A	NS	Male	55	-Pretreatment<br>- Dabrafenib clinical partial response

CNR2 mutations

Mutation	Patient number	Cancer type	Gender	Age	Other demographics
G148*	DU-145	Prostate	Male	N/A	
W172*	PD36792a	Skin	Male	72	
W172*	TCGA-Z2-A8RT-06	Skin	Female	42	
Y207*	CHG-97T	Liver	Male	76	
W317*	C000-RCWM1J	Skin	N/A	N/A	-Central European
E330*	AS-16	Soft tissue	Male	86.07	-Chinese
I346Sfs*10	1117	Urinary tract	Male	60	-Ex smoker
